# Ultra-sensitive nitrate-ion detection via transconductance-enhanced graphene ion-sensitive field-effect transistors

**DOI:** 10.1038/s41378-024-00768-4

**Published:** 2024-09-27

**Authors:** Yingming Xu, Peng Zhou, Terrence Simon, Tianhong Cui

**Affiliations:** Univeristy of Minnesota, 111 Church Street SE, Minneapolis, Minnesota 55455 US

**Keywords:** Environmental, health and safety issues, Electrical and electronic engineering

## Abstract

Current potentiometric sensing methods are limited to detecting nitrate at parts-per-billion (sub-micromolar) concentrations, and there are no existing potentiometric chemical sensors with ultralow detection limits below the parts-per-trillion (picomolar) level. To address these challenges, we integrate interdigital graphene ion-sensitive field-effect transistors (ISFETs) with a nitrate ion-sensitive membrane (ISM). The work aims to maximize nitrate ion transport through the nitrate ISM, while achieving high device transconductance by evaluating graphene layer thickness, optimizing channel width-to-length ratio (*R*_WL_), and enlarging total sensing area. The captured nitrate ions by the nitrate ISM induce surface potential changes that are transduced into electrical signals by graphene, manifested as the Dirac point shifts. The device exhibits Nernst response behavior under ultralow concentrations, achieving a sensitivity of 28 mV/decade and establishing a record low limit of detection of 0.041 ppt (4.8 × 10^−13^ M). Additionally, the sensor showed a wide linear detection range from 0.1 ppt (1.2 × 10^−12^ M) to 100 ppm (1.2 × 10^−3^ M). Furthermore, successful detection of nitrate in tap and snow water was demonstrated with high accuracy, indicating promising applications to drinking water safety and environmental water quality control.

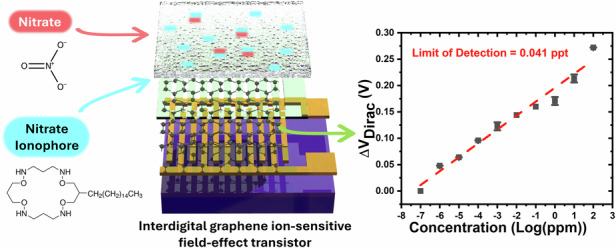

## Introduction

Detection of nitrate ions is of vital importance to human and ecological systems, as elevated nitrate levels can cause methemoglobinemia^1^, form carcinogenic N-nitroso compounds^2^, and lead to eutrophication^3^. Addressing the challenges posed by nitrate contamination requires reliable and sensitive detection methods for accurate monitoring and assessment. Recent advancements in analytical techniques, including spectrometry^4^, chromatography^5^, biosensors^6^, and electrochemical methods^7,8^, have facilitated rapid and precise detection of nitrate contaminations in various environmental samples. Among these techniques, potentiometric sensors, particularly solid-state sensors such as ion-sensitive field-effect transistors (ISFETs), offer attractive characteristics. ISFETs are low-cost, compact, rapid, ease of integration with standard electronics, real-time and on-site detection^9,10^.

Unlike metal-oxide-field-effect transistors (MOSFETs), solution-gated ISFETs utilize a solution gate consisting of a reference electrode coupled to an ion-sensitive-membrane (ISM), either in the form of glass or an ionophore complex, through an electrolyte solution^11^. As target analytes selectively bind to the ISM, the surface potential and conductance of the sensing channel are modulated^12,13^, following Nernst’s law^14^. Nitrate-sensitive ISFETs have been developed using various materials^15^ and mechanisms^16^ to enhance gate capacitance^17^ and sensing area^18^. While classical ISFETs, such as silicon-based ISFETs, have been utilized for nitrate measurement^19^, their detection limits are limited to sub-micromolar or higher^20^ due to factors such as restricted sensing area, challenges in surface functionalization, and lower carrier mobility. To overcome these limitations and enhance performance for nitrate sensing, alternative channel materials and structures have been explored, including organic ISFETs^21^ and zinc oxide nanorod ISFETs^22^ integrated with nitrate reductase. Previous studies have highlighted the importance of sensing channels with high surface-to-volume ratios, high charge carrier mobilities, large channel areas^23,24^, and high channel capacitances, all contributing to improving the detection limit and linear ranges of detection^15^.

Among these choices, graphene has emerged as a promising sensing material for nitrate detection when integrated with ISFETs^25,26^. Graphene offers several advantages, including an unprecedented charge carrier mobility of 7,000 cm²V^–^¹s^–^¹, rapid response time, ease of functionalization, high chemical stability, and high mechanical flexibility^27^. Although graphene-based ISFETs for nitrate sensing^28–30^ have shown improved detection limits and linear ranges as summarized in Table S1, the achievement of ultra-low detection of nitrate is still not well realized. Interdigital electrode design has been demonstrated to enhance signal-to-noise ratio and electron transfer between electrodes^31,32^. For graphene field-effect transistors, interdigital electrodes offer a large sensing area, high conductance, high gate capacitance, and reduced flicker noise. They have been utilized for biological^33–36^ and chemical^37^ sensing to enhance sensitivity, with detection limits in the sub-picomolar range, as summarized in Table S2. To date, an interdigital graphene-based ISFET that exhibits an ultra-low detection limit and a large linear range for nitrate sensing has not been demonstrated, and the full potential of graphene in this context has not been fully explored.

In this work, we propose ultra-sensitive graphene ISFETs for nitrate detection with a wide linear detection range. We fabricated interdigital graphene ISFETs and synthesized a nitrate ISM with a nitrate ionophore as the selective sensing layer. The thickness of the nitrate ISM was optimized to facilitate higher ion transfer. Device transconductance was studied, including investigations into graphene channel thickness and channel width-to-length ratio (*R*_WL_), to achieve an ultra-low detection limit. A linear relationship between transconductance and device sensitivity over a wide nitrate concentration range was observed. Consequently, we achieved a Nernst response ranging from 1.2 × 10^−12^ M to 1.2 × 10^−3^ M and a record low detection limit of 4.8 × 10^−13^ M. The sensor also exhibited excellent selectivity over Cl^−^, CO_3_^2−^, PO_4_^3−^, SO_4_^2−^, SO_3_^2−^, and Na^+^ interfering ions. Nitrate levels are measured in tap and snow water with high accuracy and reproducibility. This study offers a highly effective graphene ISFET platform that can be utilized for sensitive detection of aqueous nitrate pollutants.

## Results and discussion

### Device design

**Fig. 1:**
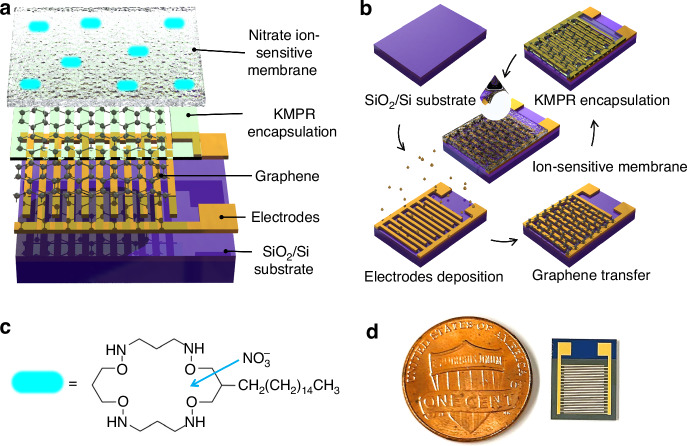
Structure and fabrication of graphene ion-sensitive field-effect transistors (ISFETs). **a** Schematic illustration of the structure of the devices. **b** Fabrication flow chart. Preparation of SiO2 substrate. E-beam deposition of Cr/Au source-drain electrodes. CVD graphene transfer. Device encapsulation via KMPR negative photoresist. Device dicing and a nitrate ion sensitive membrane deposition. **c** Chemical structure of nitrate ionophore. **d** Optical image of an individual graphene ISFET beside of one cent coin

The structure of graphene ISFETs is schematically depicted in Fig. 1a, while device fabrication is illustrated in Fig. 1b. Briefly, gold source-drain electrodes are first deposited onto the SiO_2_ wafer. After graphene transfer, oxygen plasma is utilized to pattern the graphene channels. Devices are encapsulated via KMPR photoresist to isolate the gold electrodes and define the sensing channel geometry. The wafer is diced into individual devices 9.5 mm × 12 mm, as shown in Fig. 1d and Fig. S1a. Contact wires are subsequently attached to the individual devices, which are then packaged using resin epoxy, as depicted in Fig. S1b. The nitrate ISM, utilizing a nitrate ionophore, as shown in Fig. 1c, is synthesized and deposited onto the devices. Optical images of the devices before and after the nitrate ISM deposition are presented in Fig. S1c.

### Modeling and sensing mechanism

Nitrate ions selectively bind with nitrate ionophores in the ISM, forming coordination complexes with the crown ether structure of the ionophore^38,39^, as shown in Fig. 1c. This interaction induces a potential, $E$, between the bulk electrolyte solution and the ISM that is linearly proportional to the nitrate concentration, as described by the Nernst equation shown in Eq. (1)^40^:

1 $$E={E}_{i}^{0}+\frac{{RT}}{{z}_{i}F}\mathrm{ln}{a}_{i}$$

where ${E}_{i}^{0}$ is a constant potential for analyte $i$, *R* is the universal gas constant, $T$ is temperature in Kelvin, ${z}_{i}$ is the ionic charge of analyte, $F$ is Faraday’s constant, and ${a}_{i}$ is the ionic activity, which is the concentration of the analyte ions. As the concentration of nitrate in the bulk solution changes, nitrate concentration within the membrane is simultaneously modified and the surface potential, ${\psi }_{0}$, of graphene is also changed. This change in the surface potential shifts the Fermi level, ${E}_{F}$, of graphene, as described in Eq. (2)^18^:

2 $$\varDelta {E}_{F}=\,q\frac{{C}_{g}}{{C}_{q}+{C}_{g}}\varDelta {V}_{{gate}}+q\varDelta \psi_{0}$$

where $q$ is the electric charge, ${C}_{g}$ is the geometrical gate capacitance, ${C}_{q}$ is the quantum capacitance of graphene, and ${V}_{{gate}}$ is the gate potential. For the same gate potential, the shift in Fermi level is reduced to $\varDelta {E}_{F}={q}\varDelta {\psi }_{0}$. The binding activities of nitrate-ionophore induce changes in Fermi level, causing an imbalance between the electron and hole carriers in the graphene. This imbalance is quantified by the shift in the Dirac point, which can be determined from the ${I}_{{ds}}$- ${V}_{g}$ curve. We employed the analysis of Dirac point shift, ${\varDelta V}_{{Dirac}}$, to accurately reflect alterations in nitrate concentration due to its direct correlation.

To capture the change in graphene surface potential, $\varDelta {\psi }_{0}$ or ${\varDelta V}_{{Dirac}}$, especially under ultra-low concentrations of nitrate, we designed graphene ISFETs with high transconductance, ${g}_{m}$, as described in Eq. (3)^41^:

3 $${g}_{m}=\frac{\partial {I}_{{ds}}}{\partial {V}_{g}}\,=\,\frac{W}{L}\mu {C}_{{TG}}{V}_{{ds}}$$

where *W* and *L* are the width and length of the channel, $\mu$ is the charge carrier mobility of graphene, and ${C}_{{TG}}$ is the total gate capacitance, which consists of graphene quantum capacitance, ${C}_{q}$, and geometrical gate capacitance, ${C}_{g}$. Significant enhancement in ${g}_{m}$ is achieved by applying sensing channels with high *R*_*WL*_ ${(R}_{{WL}}=\frac{W}{L})$. In addition, it is necessary to enlarge the total channel area^42^. Therefore, multiple channels were incorporated on the graphene ISFETs. Moreover, the thickness of graphene channel should be considered as the charge carrier mobility can vary significantly^43^. Finally, a relationship between ${\varDelta V}_{{Dirac}}$ and the transconductance is derived, as shown in Eq. S(1). As the transconductance increases, the shift in ${V}_{{Dirac}}$ increases.

### Graphene ISFET characterization

**Fig. 2:**
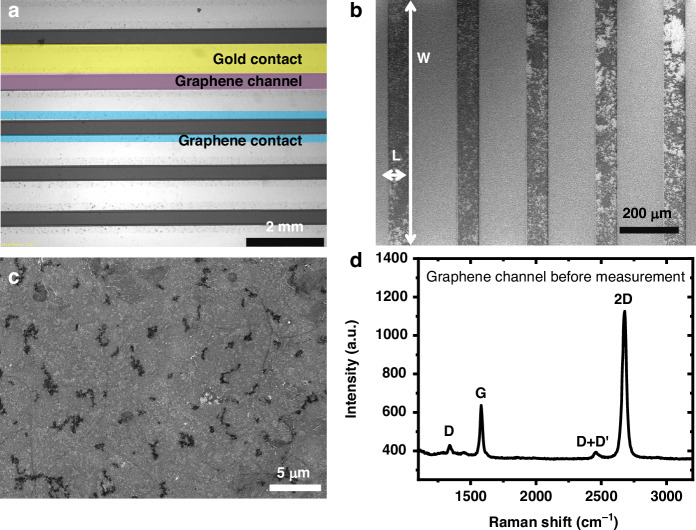
Characterization of graphene ISFET. **a** An optical image of the graphene ISFET with a 2 mm scale bar. **b** Zoomed out and **c** zoomed in images of field emission gun scanning electron microscopy measurements of graphene channels with 200 μm and 5 μm scale bar, respectively. **d** Confocal Raman spectroscopy measurement of graphene on SiO_2_ wafer

An optical image of the device is shown in Fig. 2a, where the yellow shade, purple shade, and blue shade represent the gold source-drain electrode, graphene sensing channel, and graphene-gold contact, respectively. The graphene sensing channels are further evaluated by a field emission gun scanning electron microscope, where continuous and clean channels are observed, as shown in Fig. 2b. Figure 2c indicates the transferred graphene on *SiO*_*2*_ with nucleation sites. The graphene sensing channels are characterized by confocal Raman spectroscopy, as depicted in Fig. 2d. The D, G, and 2D peaks are found at 1341 cm^−1^, 1581 cm^−1^, and 2677 cm^−1^, respectively. The peak intensity ratio of D/G is 0.15, and the G/2D peak ratio is 0.34, indicating high-quality monolayer graphene with a low density of defects and disorders in the graphene lattice. The graphene channels are also characterized after the nitrate measurement, as shown in Fig. S2a, where increases in peaks in D, D’, 2D, and D + G are observed due to the increased defect and disorder density caused by membrane removal and contamination after measurement. Moreover, regions of graphene on the gold electrodes after transfer are etched by oxygen plasma. All areas of the gold electrodes are encapsulated by KMPR. The etched regions of graphene are characterized by Raman spectroscopy to ensure full passivation. As shown in Fig. S2b, near unity ratios of D/G peaks are observed after plasma etching, due to the remaining graphene residue after the oxidation process which introduces defects and functional groups.

### Graphene ISFET electrical characteristics

**Fig. 3:**
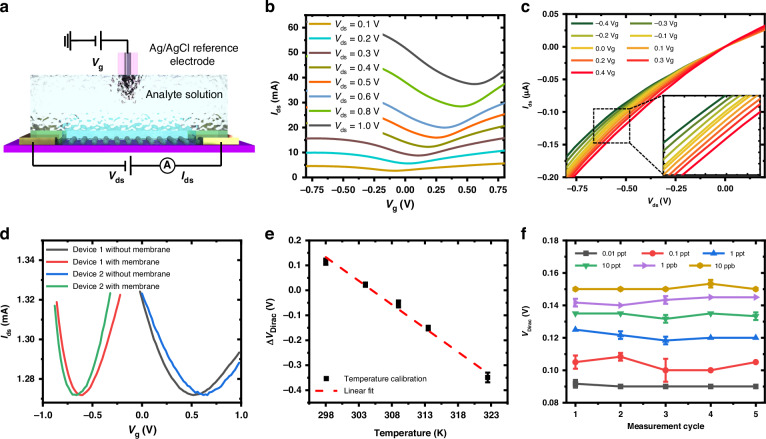
Measurement setup and electrical characteristics of graphene ISFET. **a** Measurement setup of graphene ISFET. I-V characteristics of graphene ISFET with **b** varying drain-source potential, **c** gate potential, and **d** before and after ISM deposition. **e** Graphene ISFET temperature characteristics. **f** Measurement stabilization

The measurement setup of the graphene ISFET is illustrated in Fig. S3a. The change in the Dirac point with respect to the zero-concentration nitrate ion is recorded to indicate the change in ionic concentration, as shown in Fig. S3b. The transfer characteristics of the solution-gated graphene ISFET at eight different drain-source bias potentials are shown in Fig. 3b, where the positive and negative *V*_*g*_ regions represent electron and hole transport, respectively. The output characteristics of the graphene ISFET are shown in Fig. 3c, with *V*_*g*_ varying from −0.4 V to 0.4 V, and the drain-source current is shown to be modulated by *V*_*g*_.

The graphene ISFETs with multilayer thickness channels are tested in deionized water before and after the deposition of the nitrate ISM. The *I*_*ds*_*–V*_*g*_ transfer curves are recorded as shown in Fig. 3d. The Dirac points, *V*_*Dirac*_, of the as-fabricated graphene ISFETs are positive between 0.5 V and 0.7 V, indicating the negatively charged surface residue of PMMA on the graphene after fabrication. After the deposition of the ISM, *V*_*Dirac*_ shows negative potentials with magnitudes between −0.75 V and −0.60 V due to the positively charged nitrate ISM.

With the nitrate ISM, the *V*_*Dirac*_ responses linearly with the temperature, as predicted from Nernst Eq. (1), where the potential across the ISM, *E*, is linearly proportional to temperature in Kelvin, in agreement with our experimental results as shown in Fig. 3e. To minimize the temperature effect, we control the temperature to 25 °C.

During measurements, five measurement cycles were conducted, and each consists of thirty *I*_*ds*_*–V*_*g*_ scans. The total detection time is 12 min. The last five scans from each measurement cycle are collected and averaged to serve as one measurement point. As shown in Fig. 3f, measurement points taken from five measurement cycles are plotted against *V*_*Dirac*_. Consistent and stable measurement points are obtained at different concentrations from 0.01 ppt to 10 ppb indicating high measurement stability.

### Graphene ISFET parameter optimization

The multilayer and monolayer graphene are first compared. The multilayer graphene ISFETs with 0.5 mm wide and 0.1 mm long channels, and spin coated nitrate ISM under 4500 RPM were tested in *NaNO*_*3*_ solutions with concentrations ranging from 1 ppb to 100 ppm. The responses are depicted in Fig. S4a, where uniform shifts in the transfer curve are observed. As nitrate binds to the membrane, it induces a positive doping effect on the underlying graphene channels. Consequently, positive *ΔV*_*Dirac*_ are observed, indicating an increase in the number of hole carriers, leading to a decrease in the *I*_*ds*_^44,45^. The gate potential required to attain charge neutrality, at which *I*_*ds*_ is at a minimum, is extracted and plotted against the logarithm scale of nitrate concentration, as depicted by the black curve in Fig. S4c. The multilayer graphene ISFET successfully detects nitrate ions at a concentration of 1 ppb, with a sensitivity of 30.4 mV/decade. Monolayer graphene ISFETs with the same device design and nitrate ISM were tested under identical conditions. As illustrated in Fig. S4b, the *I*_*ds*_*–V*_*g*_ transfer curve shifts positively with increasing nitrate concentrations. The *ΔV*_*Dirac*_ is extracted and plotted against nitrate concentration, as shown by the red curve in Fig. S4c. The detection limit is determined to be 0.1 ppt due to significantly enhanced charge carrier mobility and transconductance.

**Fig. 4:**
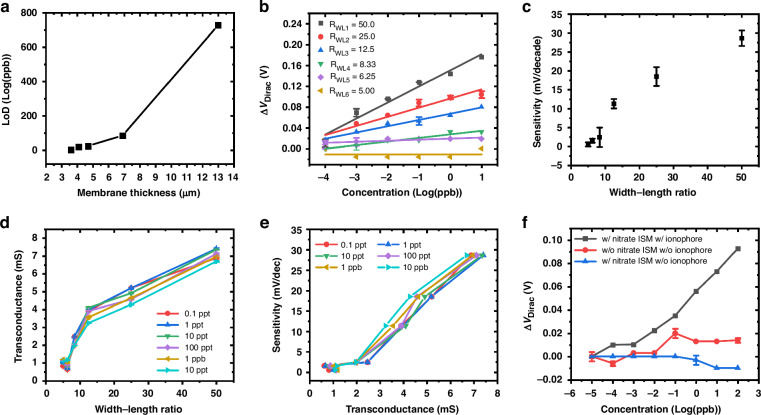
Graphene ISFET parameter optimization. **a** Limits of detection of graphene ISFETs with respect to ion-sensitive-membrane thickness. **b** Dirac point shifts under nitrate concentrations from 0.1 ppt to 10 ppb with R_WL_ from 5 to 50. **c** ISFET sensitivity change as a function of R_WL_ for nitrate concentrations from 0.1 ppt to 10 ppb. **d** ISFET transconductances as functions of R_WL_ with nitrate concentrations from 0.1 ppt to 10 ppb. **e** ISFET sensitivity as a function of transconductance under nitrate concentrations from 0.1 ppt to 10 ppb. **f** Measurements with nitrates of different ISM compositions

The influence of ISM thickness is then investigated. Binding activities between nitrate ions and the nitrate ionophore modulate the surface potential of the graphene. Therefore, the thickness of the ISM directly impacts the detection limit of the ISFET, with a thinner membrane facilitating faster and more efficient nitrate diffusion and transportation to the graphene sensing layer. This hypothesis is validated by detecting nitrate ions with different ISM thicknesses. Utilizing a simpler transfer process of multilayer graphene with thermal release tape, the study of membrane thickness is conducted with multilayer graphene ISFETs and the findings are extrapolated to monolayer graphene ISFET behavior. The graphene sensing channels are 5 mm wide and 0.1 mm long. Devices with various ISM thicknesses are tested in a *NaNO*_*3*_ solution with concentrations ranging from 1 ppb to 100 ppm. The *I*_*ds*_*–V*_*g*_ transfer curves are presented in Fig. S5a, c, e, g, i, while *ΔV*_*Dirac*_ values are plotted in Fig. S5b, d, f, h, j. The thicknesses of the nitrate ISMs are measured by a surface profiler and recorded as 13.0 μm, 6.9 μm, 5.8 μm, 4.1 μm, and 3.6 μm. They are fabricated with spin coating speeds of 1000, 2000 RPM, 3000 RPM, 4000 RPM, and 4500 RPM, respectively. An increasing trend in the detection limit is observed as the membrane thickness increases, as illustrated in Fig. 4a, consistent with the hypothesis. We observed no significant reduction in thickness with higher spin coating speeds beyond 4500 RPM, attributable to the rapid drying of the ISM and its high viscosity. Moreover, reduced thickness negatively impacts the mechanical stability of the device. Therefore, a membrane thickness of 3.6 μm under a 4500 RPM spin coating speed is selected as the optimal value for subsequent studies.

The graphene sensing channels’ width-to-length ratio, *R*_*WL*_, is investigated by varying both the channel width and length to enhance the graphene ISFET transconductance. Initially, graphene ISFETs were fabricated to compare channel widths of 2.5 mm and 5.0 mm with a channel length of 0.1 mm. As depicted in Fig. S6a, b, uniform shifts in the transfer curves are observed. *ΔV*_*Dirac*_ are plotted in Fig. S6c. Under equivalent concentrations, devices with 5.0 mm channel widths exhibit higher *ΔV*_*Dirac*_, values resulting in lower detection limits. This phenomenon can be attributed to the enlarged sensing channel area and *R*_*WL*_, which also contribute to a higher transconductance. Consequently, a channel width of 5.0 mm is maintained as a constant parameter, while the channel length is varied to modify *R*_*WL*_. Under identical measurement conditions, channel lengths ranging from 0.1 mm to 1.0 mm are assessed. Furthermore, different *R*_*WL*_ values are designated as *R*_*WL1*_ = *50*, *R*_*WL2*_ = *25*, *R*_*WL3*_ = *12.5*, *R*_*WL4*_ = *8.33*, *R*_*WL5*_ = *6.25*, and *R*_*WL6*_ = *5* for channel lengths of 0.1 mm, 0.2 mm, 0.4 mm, 0.6 mm, 0.8 mm, and 1.0 mm, respectively. The corresponding *I*_*ds*_*–V*_*g*_ transfer curves are depicted in Fig. S7a–f, while *ΔV*_*Dirac*_ values are plotted in Fig. 4b, ranging from 0.1 ppt to 10 ppb. As anticipated from Nernst’s law, the *ΔV*_*Dirac*_ values of ISFETs exhibit excellent linear relationships with increasing nitrate concentration. As illustrated in Fig. 4c, the ISFET sensitivities are identified as 0.55, 1.6, 2.4, 11, 19, and 28 mV/decade with increasing *R*_*WL*_. This enhancement can be attributed to the significant improvement in ISFET transconductance, as described in Eq. (3) and Eq. S(1). It should be noted that ISFETs with *R*_*WL*_ = *50* comprise 29 individual channels forming a total area of 14.5 mm², which results in a large gate capacitance and contributes to transconductance enhancement. As illustrated in Fig. 4d, transconductance increases linearly from nearly 1 mS to over 7 mS as *R*_*WL*_ increases from 5.0 to 50, significantly surpassing existing graphene field-effect transistor values^24,46–49^. The sensitivity is notably enhanced for ultra-low concentration detection of nitrate, and a linear relationship is evident between transconductance and sensitivity, as shown in Fig. 4e.

To exam the effectiveness of the nitrate ionophore, a control experiment was conducted. A graphene ISFET without the nitrate ISM was employed to measure nitrate ranging from 0.01 ppt to 100 ppb, revealing a small positive *ΔV*_*Dirac*_ due to nonspecific adsorption of nitrate onto the graphene channels, as indicated by the red curve in Fig. 4f. Subsequently, another device coated with ISM devoid of nitrate ionophore was measured. As demonstrated by the blue curve in Fig. 4f, the ISFET exhibits no response from 0.01 ppt to 1 ppb. The membrane is expected to obstruct nitrate ions due to the absence of binding sites without the nitrate ionophore, consistent with experimental findings. With the concentration increasing to 100 ppb, a slight negative shift is observed, likely attributable to the presence of sodium ions resulting from cation diffusion through the ion-permeable ISM. Finally, a device coated with nitrate ISM containing nitrate ionophore is tested. The device exhibits a linear Nernstian response as the concentration increases, confirming that nitrate and nitrate ionophore binding predominantly contribute to *ΔV*_*Dirac*_, as depicted by the black curve in Fig. 4f.

### Calibration, selectivity, and water sample assesment

**Fig. 5:**
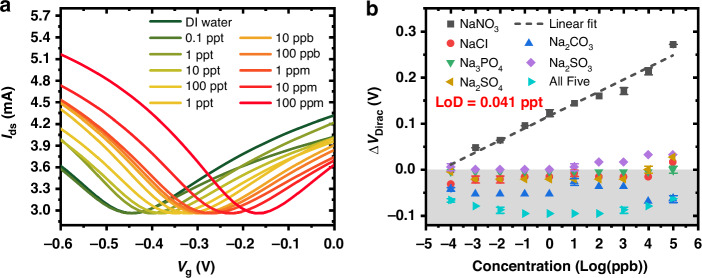
Graphene ISFET selectivity and calibration. **a** I_ds_ verses V_g_ transfer curves of nitrate ISFET with concentrations from 0.1 ppt to 100 ppm. **b** Calibration curve and Selectivity test of ISFET with different interfering cations and anions including Cl^-^, CO_3_^2−^, PO_4_^3−^, SO_4_^2−^, SO_3_^2−^, and Na^+^ with concentrations from 0.1 ppt to 100 ppm

ISFETs consisting of monolayer graphene, a 3.6 μm nitrate ISM with a channel *R*_*WL*_ of 50, and a channel area of 14.5 mm^2^ were employed to generate a calibration curve in DI water for ultra-low concentration detection of nitrate ions with a wide linear detection range. Uniform *I*_*ds*_*–V*_*g*_ transfer curves from 0.1 ppt to 100 ppm were obtained, as depicted in Fig. 5a. The *ΔV*_*Dirac*_ were extracted from the *I*_*ds*_*–V*_*g*_ curves and correlated with their corresponding concentrations of nitrate ions in the black curve in Fig. 5b. The ISFET exhibits an excellent linear relationship between the logarithmic concentrations of nitrate and the *ΔV*_*Dirac*_. A record low limit of detection was determined as 0.041 ppt, equivalent to 4.8 × 10^−13^ M of nitrate while demonstrating a wide linear range of 9 orders of magnitude between 0.1 ppt (1.2 × 10^−12^ M) and 100 ppm (1.2 × 10^−3^ M).

Graphene ISFETs with nitrate ISM were then utilized to evaluate six common interfering ions including Cl^−^, CO_3_^2−^, PO_4_^3−^, SO_4_^2−^, SO_3_^2−^, and Na^+^ with concentrations ranging from 0.1 ppt to 100 ppm. The *I*_*ds*_*–V*_*g*_ transfer curves are depicted in Fig. S8. Negligible or negative Dirac point shifts were obtained with individual or a mixture of interfering ions from 0.1 ppt to 100 ppm, as shown in Fig. 5b, indicating excellent selectivity of the devices.

The real sample measurement capability of the devices is demonstrated by measuring the changes in nitrate concentration in tap water and melted snow water. The water samples were collected from various locations at the University of Minnesota. Known concentrations of sodium nitrate were added to the water samples to mimic the nitrate level fluctuation. Five devices were used to measure the changes in the concentrations of nitrate, and the average response was utilized as the calibration curve for later reference. As shown in Fig. S9a, an increase in the detection limit is observed for real sample measurements due to high initial concentrations of nitrate in the real samples. The nitrate concentration in tap water was 5.87 ppm, and in snow water, was 0.53 ppm. according to ion chromatography analysis. According to the EPA’s drinking water standard, nitrate concentration should not exceed 10 ppm, and the limit of detection for real samples is well below the standard. The devices exhibit high accuracy at various concentrations from 10 ppb to 300 ppm with recovery value from 91% to 105% for concentrations above 10 ppm, as summarized in Table 1.

**Table 1:** Sample measurement in tap and snow water

Added [Log(ppb)]	Device measured added (tap water) [Log(ppb)]	Recovery (%)	Device measured added (snow water) [Log(ppb)]	Recovery (%)
1.00 (10 ppb)	1.10 ± 0.07	110	0.57 ± 0.56	52
2.00 (100 ppb)	2.39 ± 0.19	120	1.61 ± 0.27	67
3.00 (1 ppm)	4.07 ± 0.25	136	2.25 ± 0.69	55
4.00 (10 ppm)	4.12 ± 0.20	103	4.00 ± 0.03	97
5.00 (100 ppm)	5.05 ± 0.20	101	4.58 ± 0.24	91
5.30 (200 ppm)	5.55 ± 0.001	105	5.11 ± 0.03	92
5.48 (300 ppm)	5.78 ± 0.001	105	5.40 ± 0.09	93

## Conclusion

In this study, we have achieved the detection of nitrate ions under ultra-low concentrations with a record low limit of detection and a wide linear detection range by employing interdigital graphene ion-sensitive field-effect transistor (ISFET) design with an optimized nitrate ion-sensitive-membrane (ISM). To enable ultra-low concentration detection of nitrate, we investigated different device parameters. Specifically, we compared the effects of graphene with monolayer and multilayer channels, considered ion diffusion and transport through the nitrate ISM via precise thickness control of the membrane, and studied geometric factors of the width-to-length ratio (*R*_*WL*_) of graphene sensing channels. As a result, we accomplished significant enhancements in device transconductance of 7 mS. Consequently, ISFETs exhibit high Nernstian sensitivity of 28 mV/decade for nitrate detection under ultra-low concentrations. Graphene ISFETs with significantly enhanced transconductance are utilized to calibrate nitrate concentrations. A record low limit of detection of 0.041 ppt (4.8 × 10^−13^ M) and a large linear detection range from 0.1 ppt (1.2 × 10^−12^ M) to 100 ppm (1.2 × 10^−3^ M) are achieved. The graphene ISFETs exhibit excellent selectivity to nitrate ions over Cl^−^, CO_3_^2−^, PO_4_^3−^, SO_4_^2−^, SO_3_^2−^, and Na^+^. Real sample measurements are performed in tap water and melted snow at high accuracy, meeting the EPA’s water standard for nitrate. The ISFET device shows great potential not only for nitrate detection but also offers an excellent sensing platform for the measurement of other pollutants in water over a wide range of environmental applications.

## Materials and Methods

### Graphene ISFET fabrication

Following our previous manufacturing processes^50–52^, silicon wafers 100 mm in diameter and 525 μm thick Waferpro are prepared with 2 μm wet oxide on both sides. The wafers are cleaned using a piranha solution consisting of 70% v/v sulfuric acid and 30% v/v hydrogen peroxide. Cr/Au (10 nm/100 nm) is deposited sequentially via e-beam evaporation (CHA electron beam evaporator). The electrode patterns are transferred to the Cr/Au film using a mask aligner (Karl Suss MA6) via photolithography. The metal film is then wet etched in Au etchant and Cr etchant.

Multilayer graphene on Ni is purchased from Graphene-Supermarket and transferred via thermal release tape (Graphene-Supermarket). The thermal tape is directly attached to the multilayer graphene. A tape/graphene/Ni film on a Si wafer sample (1 cm × 1 cm) is separated under deionized (DI) water and etched with a Nickel Etchant (Nickel Etchant TFG), then rinsed in DI water. The surface of the Au electrode is treated with oxygen plasma (Plasmatherm AV2-Etcher) to clean the photoresist residues and enhance the affinity between graphene and Au. The sample is pressed against the metal electrodes and treated at 100 °C to release the thermal tape.

Monolayer graphene coated with 60 nm PMMA on 18 μm thick poly-crystalline Cu foil is purchased from Graphenea. PMMA/graphene/Cu is treated with oxygen plasma (Plasmatherm AV2-Etcher) to remove the undesired graphene on the back side of the Cu. The Cu foil is then etched by Cu etchant (415 Ferric Chloride Copper Etchant), and graphene with PMMA support is transferred to DI water three times for cleaning and scooped out with oxygen plasma treated Au on SiO_2_ substrate. After overnight drying at room temperature, the PMMA is removed with Acetone.

Multilayer and monolayer graphene are patterned and etched to form the desired channel width via photolithography (Karl Suss MA6) and oxygen plasma (Plasmatherm AV2-Etcher). Then, devices are encapsulated by KMPR negative photoresist via photolithography (Karl Suss MA6). The wafer is diced into individual ISFETs by a dicing saw (Dicing Saw DAC 552). The nitrate ion-sensitive membrane is deposited by spin coating (CEE-1 spinner) for 60 s with different coating speeds and the devices are sealed by resin epoxy after attaching contact wires.

All mentioned equipment is offered by the Minnesota Nano Center (MNC). The fabrication is performed in a class 100 cleanroom from the MNC.

### Nitrate ISM synthesis

Nitrate ISM is synthesized by mixing 25 mg of nitrate ionophore (Nitrate Ionophore VI) with 10 mg of lipophilic salt tridodecylmethylammonium chloride (TDMAC), 330 mg of poly (vinyl chloride) (PVC), 660 mg of dioctyl sebacate (DOS) plasticizer, and 4 mL of tetrahydrofuran (THF). All chemicals are purchased from Sigma Aldrich without further processing. The membrane solution is stirred overnight before deposition.

### Electrolyte solution preparation

Analyte solutions are prepared by dissolving anhydrous salts NaNO_3_ (≥99%), NaCl (≥99%), Na_2_CO_3_ (≥99%), Na_3_PO_4_ (≥96%), Na_2_SO_4_ (≥99%), and Na_2_SO_3_ (≥98%) in DI water. All salts are purchased from Sigma Aldrich without further processing. Stock solutions with 10 g/L concentration were prepared and diluted ten times to form a range of concentrations from 0.1 ppt to 1,000 ppm. All glassware and devices are thoroughly rinsed with DI water before and after measurement.

### Device characterization

The graphene channels of ISFETs are measured by Raman spectroscopy (Witec Alpha 300R confocal Raman microscope) with YAG 532 nm laser, a FESEM (Hitachi SU8230 Field Emission Gun Scanning Electron Microscope) with an acceleration voltage of 5 kV, and an optical microscope (Olympus SZX16). The thickness of the synthesized membrane after deposition is measured by a surface profiler (Surface Profiler P7 KLA-Tencor).

### Device measurement

The graphene ISFETs are measured by submerging them into analyte solutions with various concentrations. The electrical signal including the source-drain current and Dirac point are monitored by a semiconductor analyzer (Agilent HP 4156A Precision Semiconductor Parameter Analyzer) and an electrochemical workstation (Autolab PGSTAT302N). The source-drain currents are recorded versing the gate potential from −1 to 1 V that is applied to an Ag/AgCl reference electrode with a constant source-drain bias potential of 100 mV, a scan rate of 0.4 V/s, and a resolution of 0.01 V.

## Supplementary information

Supplementary Material
