# Supplementary material for: Ultra-sensitive nitrate-ion detection via transconductance-enhanced graphene ion-sensitive field-effect transistors
Source: Microsyst Nanoeng. 2024 Sep 27;10:137. doi: 10.1038/s41378-024-00768-4 (PMC11427685; doi:10.1038/s41378-024-00768-4)
Supplement: Supplementary file 1 — Supplementary Material [file 41378_2024_768_MOESM1_ESM.docx]

**Supplementary Material**

Ultra-Sensitive Nitrate-Ion Detection via Transconductance Enhanced Graphene Ion-Sensitive Field-Effect Transistors

Yingming Xu^1^, Peng Zhou^1^, Terrence Simon^1^, Tianhong Cui^1*^

^1^Department of Mechanical Engineering, University of Minnesota, 111 Church Street SE, Minneapolis, MN 55455, USA

^*^Corresponding Author: *Tianhong Cui, Phone: 612-626-1636, E-mail: cuixx006@umn.edu

1. **Device fabrication**

The graphene ion-sensitive field-effect transistors (ISFETs) utilize wafer-scale microfabrication techniques to reduce the cost per device, as illustrated in Figure S1.a. After dicing the wafer into individual devices, contact wires are attached to the electrodes using silver conductive epoxy. Subsequently, the device is sealed with resin epoxy, as depicted in Figure S1.b. A colorless and transparent nitrate ion-sensitive membrane (ISM) is deposited onto the devices via spin coating, as shown in Figure S1.c, which illustrates the graphene ISFET before and after the deposition of the nitrate ISM.


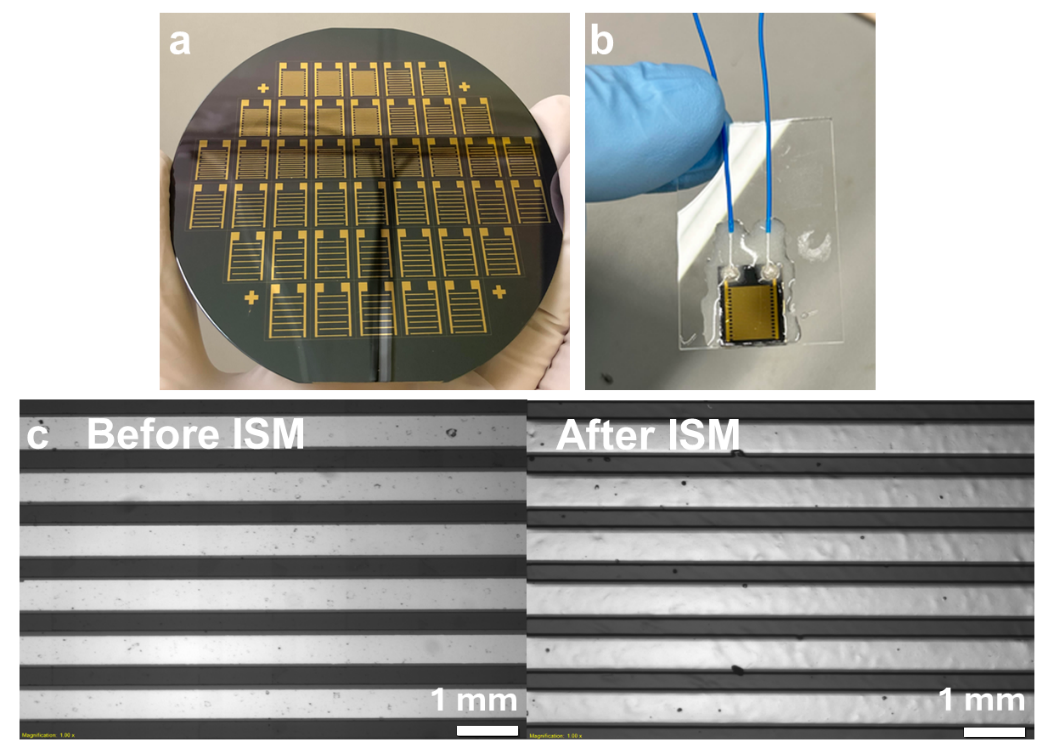


**Figure S1.** **a** Wafer scale fabrication of graphene ISFETs with different channel width to length ratios. **b** Packaged graphene ISFET. **c** Graphene ISFET channels before and c after deposition of nitrate-ion-sensitive membrane (colorless and transparent).

1. **Summary table of recent ISFETs for nitrate detection.**

The current nitrate ISFETs with a single pair of electrodes have shown limited detection limits, ranging from hundreds of millimolar to nanomolar, as summarized in Supplementary Table 1. Kim et al. developed a solution-gated graphene ISFET with a nitrate ISM consisting of a nitrate ionophore in a PVC membrane. They demonstrated a detection limit of 1.6×10^−6^ M with a linear range from 1.6×10^−6^ to 1.6×10^−3^ M [1, 5]. To further reduce the detection limit, Kim et al. fabricated graphene ISFETs on a porous anodic aluminum oxide (AAO) substrate with a nitrate ISM, achieving an improved detection limit of 8.1×10^−7^ M with a linear range from 8.1×10^−7^ to 1.6×10^−4^ M [2]. Fakih et al. applied a single-pair large-area graphene ISFET with different ionophore ISMs to monitor various nutrient ions, including nitrate. They demonstrated a detection limit of 1.0×10^−5^ M with a linear range from 1.0×10^−5^ to 2.0×10^−2^ M [4]. In addition to the nitrate ionophore ISM, other mechanisms are also utilized for nitrate detection. Liu et al. designed a graphene ISFET with a triethylamine probe molecule to enable selective detection, demonstrating a detection limit of 1.3×10^−9^ M. Bhat et al. designed a zinc oxide nanorod ISFET array for the detection of phosphate, nitrate, and potassium, showing a detection limit of 1.0×10^−7^ M with a linear range from 2.0×10^−6^ to 2.5×10^−2^ M [6].

**Supplementary table 1. Current Nitrate ISFET**


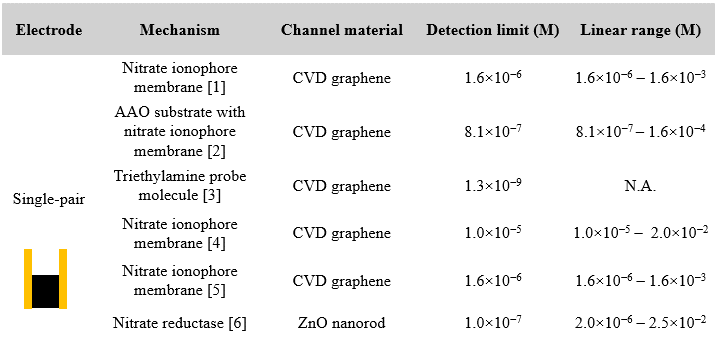


1. **Summary table of recent interdigital graphene ISFETs for biological and chemical sensing.**

Interdigital graphene ISFETs have been widely employed as biosensors and chemical sensors for ultra-low concentration detection, as summarized in Supplementary Table 2. Wang et al. designed an interdigital graphene ISFET for biotin detection, demonstrating an ultra-low detection limit of 3.7×10^−13^ M [7]. They also utilized a similar device structure for the detection of biotinylated macromolecules, achieving a detection limit of 4.0×10^−13^ M [9]. Thanh et al. developed an interdigital carbaryl graphene ISFET with a detection limit of 5.0×10^−11^ M [8]. Another graphene ISFET biosensor with an interdigital electrode design was developed by Wang et al. for neuron-specific enolase, with a detection limit of 1.1×10^−13^ M [10]. Wang et al. also developed a carboxyl graphene ISFET with an interdigital electrode, demonstrating a detection limit of 1.0×10^−9^ M [11]. Despite the extensive application of interdigital graphene ISFETs in various biological and chemical sensing contexts, interdigital nitrate graphene ISFETs with sensitive nitrate ISMs have not been previously demonstrated. We have combined the nitrate ISM with an interdigital graphene ISFET to achieve an ultra-low detection limit of 4.8×10^−13^ M, with a wide linear range from 1.2×10^−12^ to 1.2×10^-3^ M.

**
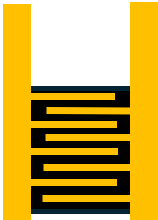
**

**Supplementary table 2. Current interdigital FET**


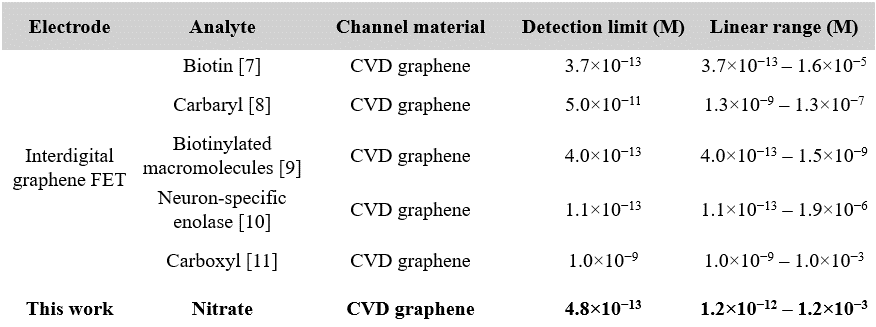


1. **Raman spectroscopy characterization of CVD monolayer graphene.**

The graphene channels are characterized using Raman spectroscopy. Following the nitrate measurement, the nitrate ISM is peeled off to expose the underlying graphene channels. As illustrated in Figure S.2a, an increase in peaks D, D’, 2D, and D+G is observed, attributed to the heightened defect and disorder density resulting from membrane removal and contamination post-measurement.

To isolate the graphene channels for nitrate sensing, the graphene on the gold electrodes, post-transfer, is passivated through oxygen plasma etching and encapsulated by KMPR photoresist. Subsequently, the passivated graphene on gold is analyzed via Raman spectroscopy. As depicted in Figure S2.b, the pristine graphene is transformed into graphene oxide after oxygen plasma treatment, as evidenced by an approximate 1:1 ratio of D to G peak. This observation indicates successful conversion of graphene into graphene oxide, if not entirely removed.


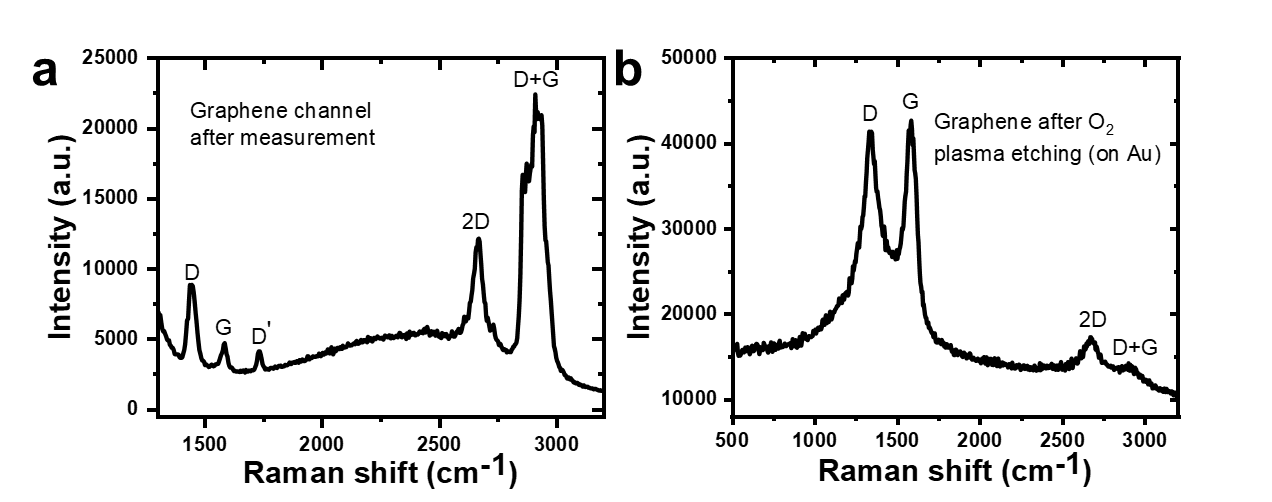


**Figure S2.** Confocal Raman spectroscopy measurement of graphene **a** after nitrate measurements (after nitrate ion-sensitive-membrane removal) and **b** graphene on gold contact after oxygen plasma etching.

1. **Graphene ISFET measurement setup, Dirac point extraction, and transconductance calculation.**

Analyte solutions are prepared by dissolving anhydrous salts NaNO3 (≥99%), NaCl (≥99%), Na2CO3 (≥99%), Na3PO4 (≥96%), Na2SO4 (≥99%), and Na2SO3 (≥98%) in DI water. All salts are purchased from Sigma Aldrich without further processing. Stock solutions with 10 g/L concentration were prepared and diluted ten times to form a range of concentrations from 0.1 ppt to 1,000 ppm. All glassware and devices are thoroughly rinsed with DI water before and after measurement.

The setup for graphene ISFET measurement is depicted in Figure S3.a. A constant bias potential of 100 mV is applied between the drain-source electrode, while the gate potential, modulated by a Ag/AgCl reference electrode, is swept. The packaged device is immersed in the analyte solution for measurement. The drain-source current, along with its corresponding gate potential, is plotted as illustrated in Figure S3.b. The charge neutrality position of graphene, known as the Dirac point, is then extracted. As anions bind to the nitrate ISM, a positive doping effect on the graphene ISFET is observed, resulting in a positive shift of the Dirac point. Furthermore, the transconductance of the device is determined by calculating the partial derivative of the drain-source current with respect to the gate potential, or by determining the slope of the linear region on the I_ds_-V_g_ curve.

**Figure S3.** **a** Graphene ISFET measurement setup. **b** demonstration of graphene Dirac point shift after doping and calculation of transconductance.

1. **Derive relationship between device transconductance and graphene ISFET Dirac point shift.**

A relationship between the transconductance and the graphene ISFET Dirac point shift is derived as shown below. As the device transcondu ctance increases, the Dirac point shift increases. Therefore, a higher sensitivity will be obtained by enhancing the device transconductance.

${\Delta\psi_{0}=C}_{3}-\frac{C_{1}}{g_{m}+C_{2}}$ (Eq. S(1))

where $C_{1}=C_{g}{R_{WL}\mu V}_{ds}\Delta V_{gate}$, $C_{2}=C_{g}{R_{WL}\mu V}_{ds}$, and $C_{3}=\frac{\Delta E_{F}}{q}$.

$\psi_{0}=-\alpha\ln10\frac{kT}{zq}\frac{{\log\left[ a \right]}_{\mathrm{membrane}}}{{\log\left[ a \right]}_{\mathrm{solution}}}=\psi_{0}^{0}+\alpha\ln10\frac{kT}{zq}{\log\left[ a \right]}_{solution}$ [4] (1)

${\Delta\psi}_{0}=\alpha\ln10\frac{kT}{zq}(\Delta log[a])$ [4] (2)

$\Delta E_{F}= q\frac{C_{g}}{C_{q}+C_{g}}\Delta V_{gate}+q\Delta\psi_{0}$ [4] (3)

$g_{m}= \frac{\partial I_{ds}}{\partial V_{g}} = \frac{W}{L}\mu C_{TG}V_{ds}$ [12] (4)

$\frac{1}{C_{TG}}=\frac{1}{C_{q}}+\frac{1}{C_{g}}$ [13] (5)

$C_{EDL}=C_{TG}=\frac{C_{q}C_{g}}{C_{q}+C_{g}}\approx C_{q} \mathrm{when} C_{q}\ll C_{g}$ [14] (6)

**Substitute (6) into (4):** $g_{m}= \frac{W}{L}\mu C_{TG}V_{ds}\approx\frac{W}{L}\mu C_{q}V_{ds}$ (7)

**Rewrite (7):** $C_{q}\approx\frac{g_{m}}{R_{WL}\mu V_{ds}}$ where $R_{WL}=\frac{W}{L}$ (7)

**Substitute (7) into (3):** $\Delta E_{F}=q\frac{C_{g}}{\frac{g_{m}}{R_{WL}\mu V_{ds}}+C_{g}}\Delta V_{gate}+q\Delta\psi_{0}$ (8)

**Define constants:** $C_{1}=C_{g}{R_{WL}\mu V}_{ds}\Delta V_{gate}$

$C_{2}=C_{g}{R_{WL}\mu V}_{ds}$

$C_{3}=\frac{\Delta E_{F}}{q}$

**Rewrite (8) with defined constants:** $C_{3}=\frac{C_{1}}{g_{m}+C_{2}}+\Delta\psi_{0}=\frac{C_{1}}{g_{m}+C_{2}}$ (9)

**Rearrange (9):** ${\Delta\psi_{0}=C}_{3}-\frac{C_{1}}{g_{m}+C_{2}}$ (10)

**Eq. S(1) is obtained as shown below.**

$${\Delta\psi_{0}=C}_{3}-\frac{C_{1}}{g_{m}+C_{2}}$$

**Definition of parameters:**

$\psi_{0}$: surface potential

$\alpha$: dimensionless sensitivity factor between 0 and 1

$k$: Boltzmann’s constant

$T$: temperature

$q$: electric charge

$z$: valency

$[a]$: activity (concentration) of the target ion

$\psi_{0}^{0}$: surface potential constant

$E_{F}$: Fermi level

$g_{m}$: transconductance

$I_{ds}$: drain source current

$V_{g}$: gate potential

$W$: width of graphene channel

$L$: length of graphene channel

$\mu$: charge carrier mobility

$C_{TG}$: total gate capacitance

$V_{ds}$: drain source bias potential

$C_{q}$: graphene quantum capacitance

$C_{g}$: geometrical gate capacitance

$C_{EDL}$: electrical double layer

$R_{WL}$: width-to-length ratio

1. **Graphene layer thickness study. Comparison between multilayer and monolayer graphene ISFET**

In order to enhance the device transconductance, graphene with varying layer thicknesses was initially investigated. Multilayer graphene was grown on nickel, while monolayer graphene was grown on copper. Fabrication of Multilayer graphene ISFETs was achieved through thermal release tape transfer, owing to its straightforward fabrication process. Given the monolayer thickness of monolayer graphene, the transfer process employed a wet PMMA support method.

Figure S4.a illustrates the Ids-Vg transfer curve of multilayer graphene ISFET, demonstrating a detection limit of 1 ppb. Upon transitioning to monolayer thickness, a notably lower detection limit of 0.1 ppt was achieved, as depicted in Figure S4.b. The extracted Dirac point shifts are presented in Figure S4.c, where the monolayer graphene ISFET exhibited higher responses under equivalent nitrate concentrations.


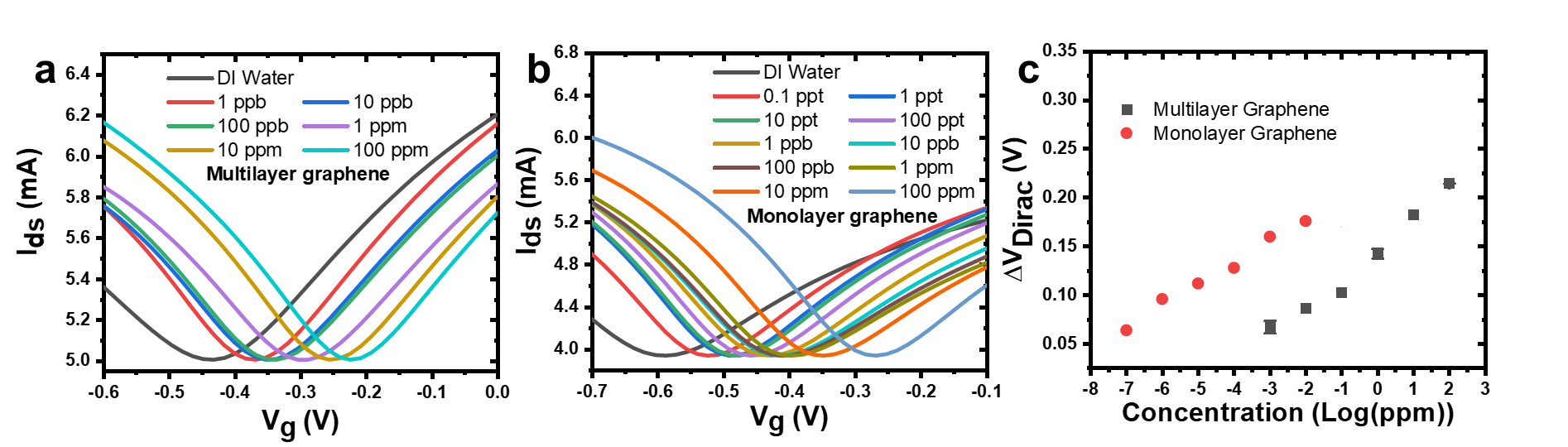


**Figure S4.** I_ds_ - V_g_ transfer curve of **a** multilayer graphene ISFET and **b** monolayer graphene ISFET. **c** Dirac point shifts of multilayer graphene ISFET (black) and monolayer graphene ISFET (red).

1. **Nitrate ISM thickness study. Multilayer graphene ISFET responses under varied ISM thickness.**

Although monolayer graphene ISFETs exhibit a lower detection limit, their fabrication process is more time-consuming and complicated compared to multilayer graphene ISFETs. Consequently, multilayer graphene ISFETs are still preferred for studying the effects of nitrate ISM thicknesses.

The nitrate ISM is deposited onto ISFETs via spin coating. Devices with various thicknesses are tested in a NaNO3 solution with concentrations ranging from 1 ppb to 100 ppm. The Ids-Vg transfer curves are presented in Figure S5.a, c, e, g, and i, while ΔV_Dirac_ values are plotted in Figure S5.b, d, f, h, and j. The thicknesses of the nitrate ISMs are measured using a surface profiler and recorded as 13 μm, 6.9 μm, 5.8 μm, 4.1 μm, and 3.6 μm, fabricated with spin coating speeds of 1000 RPM, 2000 RPM, 3000 RPM, 4000 RPM, and 4500 RPM, respectively.

As the nitrate ISM becomes thinner, the detection limit decreases. However, when the spin coating rate exceeds 4500 RPM, no significant reduction in ISM thickness is achieved. Moreover, excessively thin thickness negatively impacts the mechanical stability and selectivity of the nitrate ISM. Therefore, a thickness of 3.6 μm at a spin coating speed of 4500 RPM is selected as the optimal value for subsequent studies.


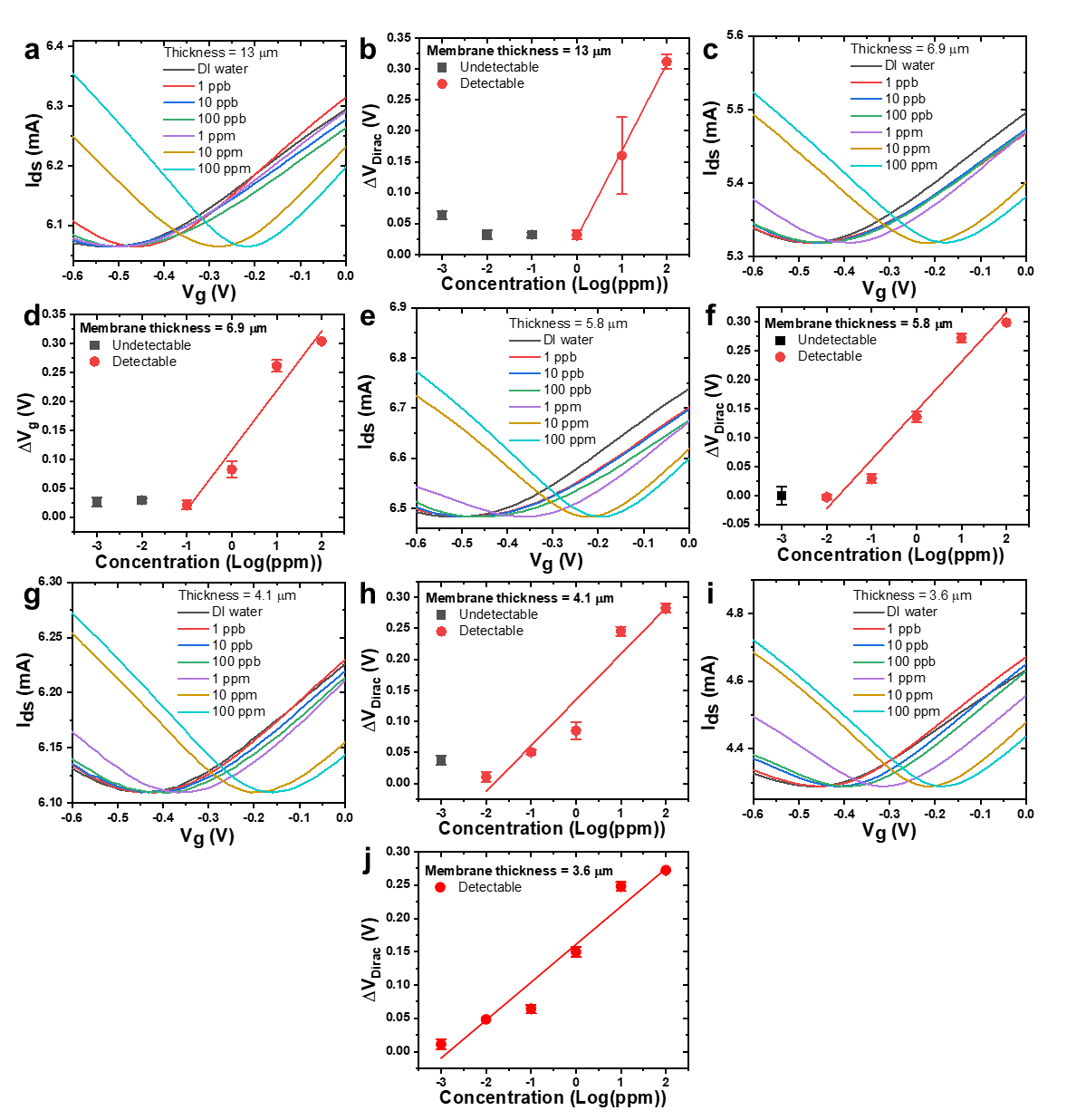


**Figure S5.** I_ds_ - V_g_ transfer curves of multilayer graphene ISFET with membrane thickness of **a** 13 μm, **c** 6.9 μm, **e** 5.8 μm, **g** 4.1 μm, and **i** 3.6 μm under concentrations from 1 ppb to 100 ppm. Dirac point shifts of multilayer graphene ISFET with membrane thickness of **b** 13 μm, **d** 6.9 μm, **f** 5.8 μm, **h** 4.1 μm, and **j** 3.6 μm under concentrations from 1 ppb to 100 ppm.

1. **Width-to-length ratio study. Multilayer graphene ISFET response comparison between 2.5 mm and 5.0 mm channel width.**

Multilayer graphene ISFETs are still being used to study the width of the sensing channels due to their straightforward fabrication process. To modify the width-to-length ratio (R_WL_) of the graphene sensing channel, the length of the channel is initially fixed, while the width is varied. Multilayer graphene ISFETs are fabricated to compare channel widths of 2.5 mm and 5.0 mm, both with a channel length of 0.1 mm.

As illustrated in Figure S6.a and b, uniform shifts in the transfer curves are observed. Dirac point shifts are extracted and plotted in Figure S6.c. Devices with a greater channel width exhibit higher ΔV_Dirac_ under equivalent concentrations, resulting in a lower detection limit due to the enhanced sensing area which also contributes to a higher transconductance.


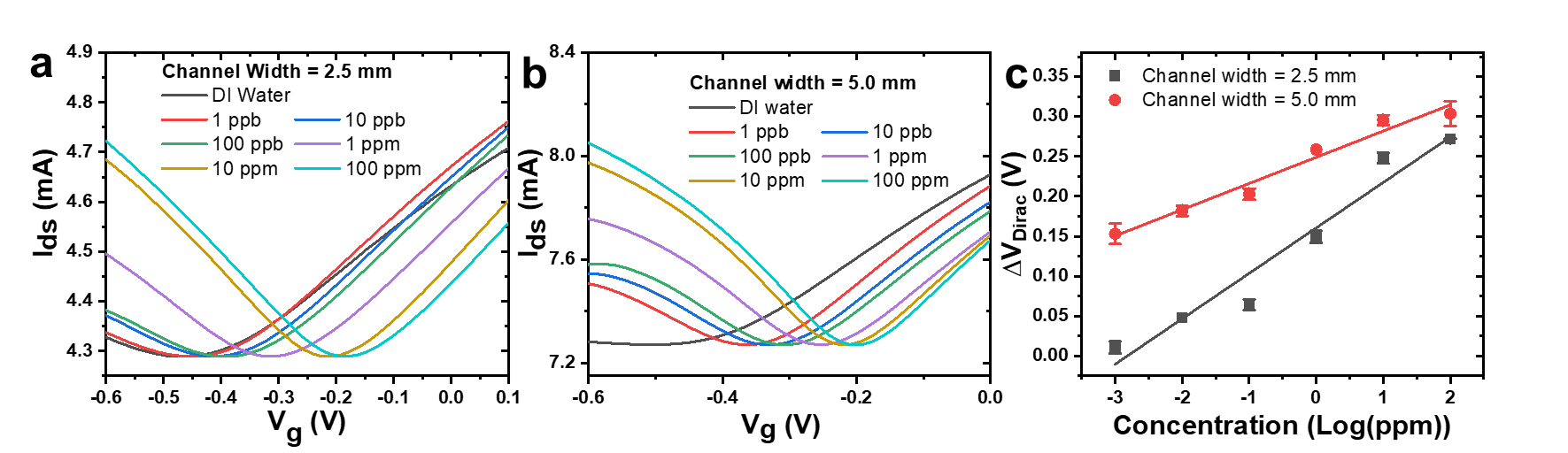


**Figure S6.** I_ds_ - V_g_ transfer curve of multilayer graphene ISFET with **a** 2.5 mm channel width and **b** 5.0 mm channel width. **c** Dirac point shifts of multilayer graphene ISFET with 2.5 mm channel width (black) and 5.0 mm channel width (red).

1. **Width-to-length ratio study. Monolayer graphene ISFET response with varied channel lengths.**

To further adjust the width-to-length ratio of the graphene sensing channels, monolayer graphene is employed as the sensing material due to its suitability for larger-scale fabrication and superior performance.


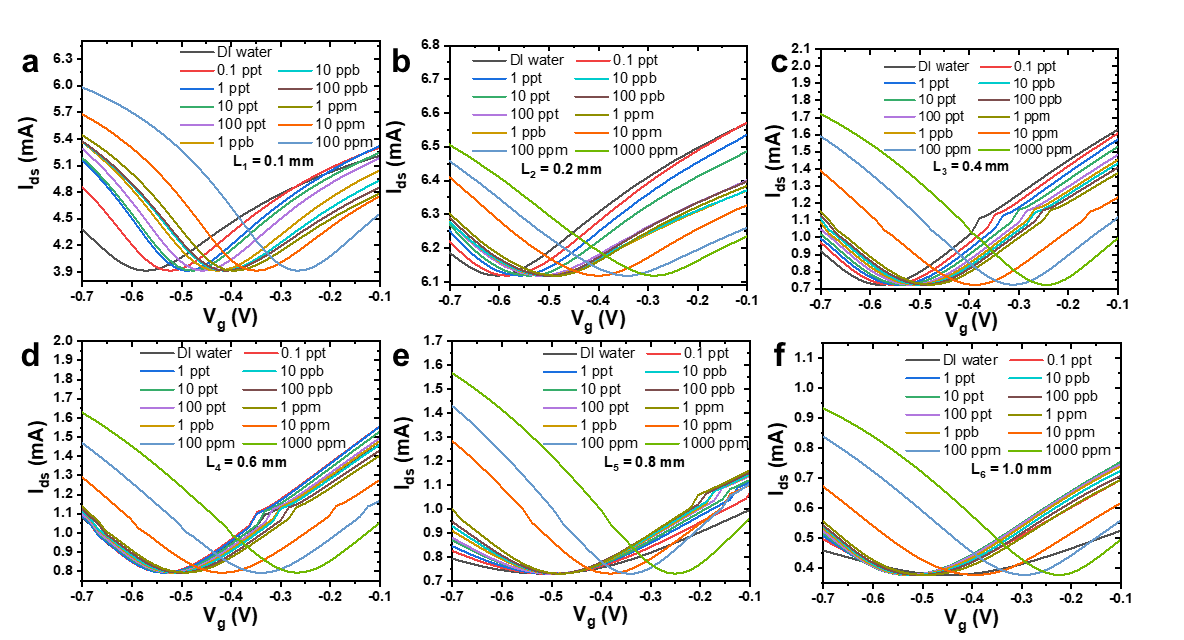


**Figure S7.** I_ds_ verses V_g_ transfer curves of monolayer graphene ISFET with channel length of **a** 0.1 mm, **b** 0.2 mm, **c** 0.4 mm, **d** 0.6 mm, **e** 0.8 mm and **f** 1.0 mm under concentration from 0.1 ppt to 1000 ppm.

A channel width of 5.0 mm is maintained as a constant parameter, while the channel length is varied to modify the Ratio of Width to Length (RWL). Under identical measurement conditions, monolayer graphene ISFETs with a 3.6 μm membrane thickness and 5.0 mm channel width are utilized, with channel lengths ranging from 0.1 mm to 1.0 mm assessed. Furthermore, different RWL values are designated as RWL1 = 50, RWL2 = 25, RWL3 = 12.5, RWL4 = 8.33, RWL5 = 6.25, and RWL6 = 5 for channel lengths of 0.1 mm, 0.2 mm, 0.4 mm, 0.6 mm, 0.8 mm, and 1.0 mm, respectively.

The corresponding Ids-Vg transfer curves are depicted in Figure S7.a, b, c, d, e, and f. The Ids-Vg curve shifts uniformly as the concentration of nitrate increases. Moreover, as the RWL decreases from 50 to 5, the detection limit decreases accordingly due to a significant reduction in transconductance.

1. **Graphene ISFET with nitrate ISM selectivity test. ISFET measurement in different interfering ions dissolved in deionized water.**

Graphene ISFETs with nitrate ISM, monolayer graphene sensing channel, and R_WL_ of 50 were utilized to evaluate six common interfering ions including Cl^-^, CO_3_^2-^, PO_4_^3-^, SO_4_^2-^, SO_3_^2-^, and Na^+^ with concentrations ranging from 0.1 ppt to 100 ppm. The I_ds_-V_g_ transfer curves are depicted in Figure S8. Negligible or negative Dirac point shifts were obtained with individual or a mixture of interfering ions from 0.1 ppt to 100 ppm, indicating excellent selectivity of the devices.


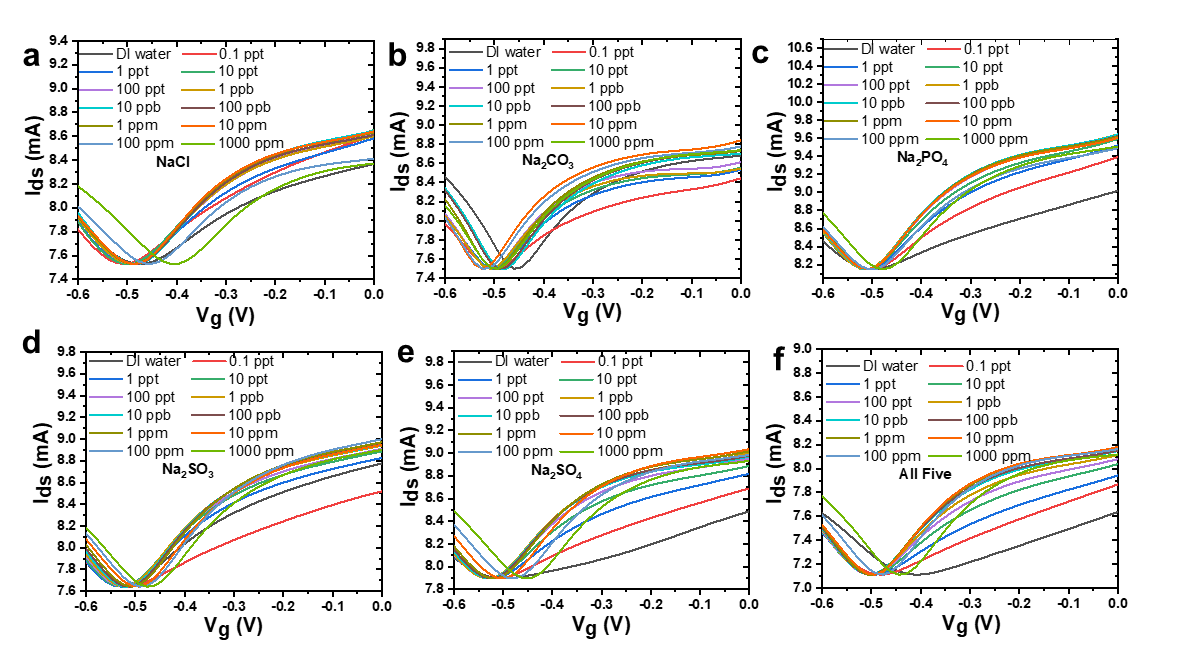


**Figure S8.** I_ds_ verses V_g_ transfer curves of monolayer graphene ISFET under interfering ions of **a** NaCl, **b** Na_2_CO_3_, **c** Na_3_PO_4_, **d** Na_2_SO_3_, **e** Na_2_SO_4_, and **f** mixture of all five-interfering ion under concentration from 0.1 ppt to 1000 ppm.

1. **Graphene ISFET real sample test. ISFET measurement in tap and snow water with different added sodium nitrate concentrations.**

The real sample measurement capability of the devices is demonstrated by measuring the changes in nitrate concentration in tap water and melted snow water. The water samples are collected from different locations within the University of Minnesota. Known concentrations of sodium nitrate are added to the water samples. Five devices were used to measure the added concentrations of nitrate, and the average response is utilized as the calibration curve for later reference. As illustrated in Figure S9.a, two linear calibration curves are obtained by linear fitting for measurements of two concentration regions, from 100 ppt to 1 ppm and from 10 ppm to 300 ppm. An increase in the detection limit is observed for real sample measurement due to highly complex interferences.


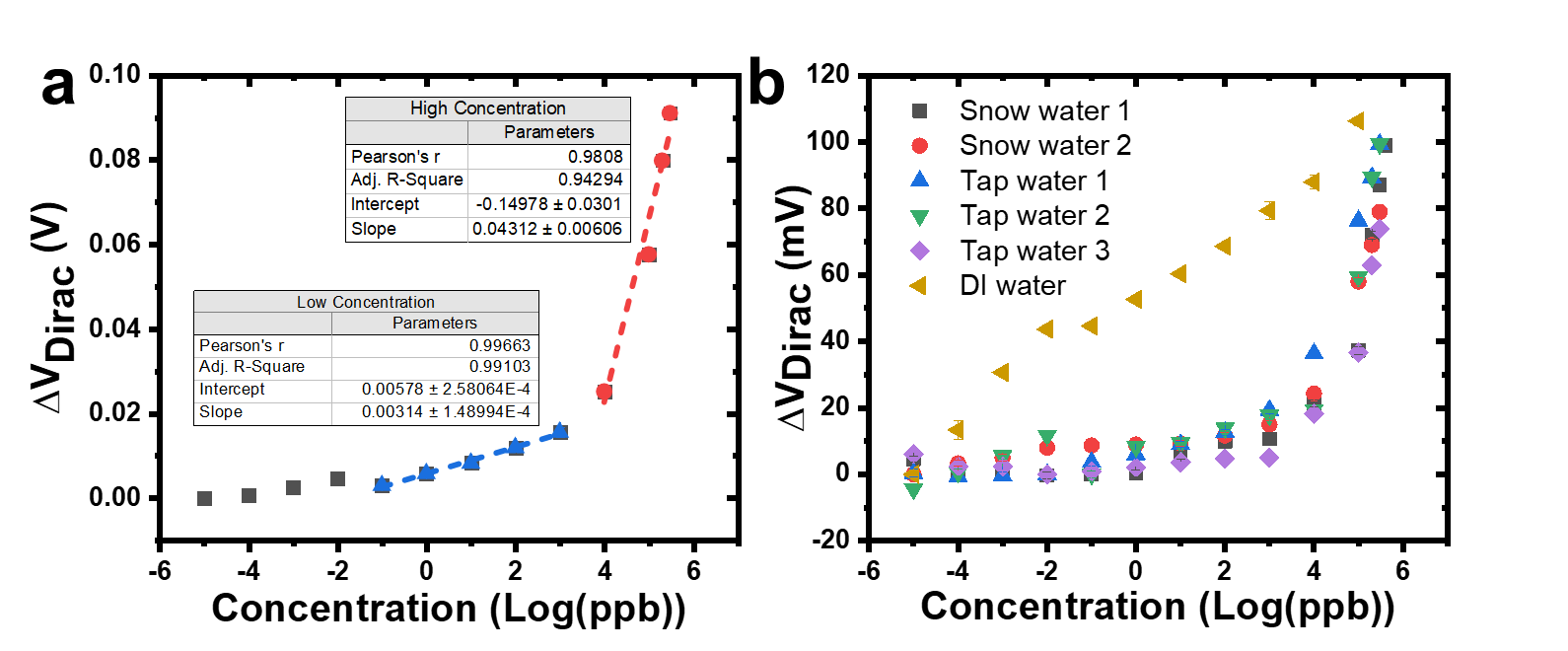


**Figure S9.** **a** Averaged calibration curve of five graphene ISFET for tap and snow water measurements with lower concentration (blue, 100 ppt to 1 ppm) and higher concentration (red, 10 ppm to 300 ppm) linear regions. **b** Tap, snow, and deionized water measurements.

1. **Supplementary reference**
2. Kim, J. et al. Solution-gated ion-sensitive field effect transistor with polymer selective membrane for nitrate detection. In *ASME International Mechanical Engineering Congress and Exposition* (Vol. 52019, p. V002T02A089). American Society of Mechanical Engineers (2018).
3. Kim, J. et al. Graphene-Based Ion Sensitive-FET Sensor With Porous Anodic Aluminum Oxide Substrate for Nitrate Detection. *Journal of Microelectromechanical Systems*, *29*(5), 966-971 (2020).
4. Liu, J. et al. Ultrasensitive graphene sensor for nitrate detection using triethylamine as a probe molecule. *Microchemical Journal*, *183*, 108043 (2022).
5. Fakih, I. et al. Selective ion sensing with high resolution large area graphene field effect transistor arrays. *Nature communications*, *11*(1), 3226 (2020).
6. Kim, J. et al. Solution-gated nitrate sensitive field effect transistor with hybrid film: CVD graphene/polymer selective membrane. *Organic Electronics*, *78*, 105551 (2020).
7. Bhat, K. S. et al. High performance chemical sensor with field-effect transistors array for selective detection of multiple ions. Chemical Engineering Journal, 417, 128064 (2021).
8. Wang, S. et al. Graphene field-effect transistor biosensor for detection of biotin with ultrahigh sensitivity and specificity. *Biosensors and Bioelectronics*, *165*, 112363 (2020).
9. Thanh, C. T. et al. An interdigitated ISFET-type sensor based on LPCVD grown graphene for ultrasensitive detection of carbaryl. *Sensors and Actuators B: Chemical*, *260*, 78-85 (2018).
10. Wang, S. et al. Avidin–biotin technology in gold nanoparticle-decorated graphene field effect transistors for detection of biotinylated macromolecules with ultrahigh sensitivity and specificity. *ACS omega*, *5*(46), 30037-30046 (2020).
11. Wang, X. et al. Highly sensitive biosensor for neuron-specific enolase detection with Bovine-serum-albumin doped graphene field-effect transistor. *IEEE Sensors Journal* (2023).
12. Wang, Z. et al. Free radical sensors based on inner-cutting graphene field-effect transistors. *Nature communications*, *10*(1), 1544 (2019).
13. Xu, H. et al. Top-gated graphene field-effect transistors with high normalized transconductance and designable dirac point voltage. *ACS nano*, *5*(6), 5031-5037 (2011).
14. Shepard, K. L. et al. Characterization and modeling of graphene field-effect devices. In *2008 IEEE/ACM International Conference on Computer-Aided Design* (pp. 406-411). IEEE (2008).
15. Uesugi, E. et al. Electric double-layer capacitance between an ionic liquid and few-layer graphene. *Scientific reports*, *3*(1), 1595 (2013).
